# Postoperative Infections Following Open Tibia Fractures: A Retrospective Study Evaluating Incidence and Prognostic Factors at a Major Trauma Centre

**DOI:** 10.7759/cureus.97995

**Published:** 2025-11-28

**Authors:** Ruqaiya Al-Habsi

**Affiliations:** 1 Surgery, The Royal London Hospital, London, GBR

**Keywords:** fracture-related infection, fri, gustilo anderson classification, lower extremity trauma, open lower leg fracture, open tibia fracture

## Abstract

Background

Open fractures are common, high-risk injuries in which the fracture site communicates with the external environment, predisposing patients to postoperative fracture-related infection (FRI). These infections can lead to prolonged hospital stay, additional surgery and long-term morbidity.

Objectives

To determine the incidence of FRI following open tibia fractures at a single major trauma centre and explore potential prognostic factors, including timing of antibiotic administration, timing of initial debridement, and definitive skeletal and soft-tissue management.

Methodology

This was a five-year retrospective observational study at a UK major trauma centre. Adult patients (≥18 years) with open tibia fractures within the inclusion period were identified from trauma meeting records and electronic patient records. Patients who received their initial management at other hospitals were excluded. Data collected included patient demographics, injury characteristics, timing of antibiotic administration, timing and type of operative fixation, soft-tissue management and occurrence of postoperative infection.

Results

A total of 440 patients with open tibia fractures presented during the study period; 340 met the inclusion criteria and were analysed. Postoperative infection occurred in 33/340 cases (9.7%). The mean age of infected patients was 45 years, and most were male. Intravenous antibiotics were administered within three hours of injury in 32/33 (97%) infected cases. Overall, 9/33 (27%) infections were superficial, and 13/33 (39%) were deep, with seven patients requiring further procedures for removal of metalwork.

Conclusions

In this single-centre cohort, postoperative FRI occurred in approximately one in 10 patients with open tibia fractures. Earlier administration of antibiotics, timely initial debridement and primary soft-tissue closure were associated with lower observed infection rates and may represent key targets for optimisation of care pathways.

## Introduction

Open fractures represent a major clinical challenge due to their combination of bony injury and soft tissue disruption, which exposes the fracture site or fracture haematoma to the external environment [1]. Even when skin wounds do not directly overlie the fracture, any nearby breach in soft tissue continuity may permit contamination, and fractures should, therefore, be treated as open until proven otherwise [1]. Open fractures should also be considered in cases where the overlying skin wound does not directly lie over the fracture but is within the same anatomical segment, as previously described in the literature [2]. These injuries are associated with substantial morbidity, particularly when infection occurs. Fracture-related infection (FRI) may present early with erythema, swelling, warmth and pain, and at later stages can contribute to delayed or impaired fracture healing [3]. The risk of infection varies according to injury severity and mechanism, with open tibia fractures demonstrating some of the highest reported rates among all long-bone injuries [4]. 

Standardised diagnostic criteria for FRI have been developed through international expert consensus, distinguishing confirmatory clinical features such as purulence or sinus formation from suggestive features that include pyrexia, soft-tissue inflammation and raised inflammatory markers [5]. These definitions have improved consistency in reporting and diagnosis, yet the factors contributing to infection in open tibia fractures remain multifactorial and centre-dependent. Early management, including antibiotic timing, debridement timing and soft tissue coverage method, plays a key role but can vary due to logistical, operative and patient-specific considerations. 

Open tibia fractures remain one of the. Most commonly encountered complex I injuries within major trauma systems. Evaluating local patterns of infection and identifying modifiable elements within the early management pathway are therefore essential to improving outcomes, optimising resource use and supporting adherence to established best practice across specialist centres. 

Study aim

This retrospective observational study aimed to determine the incidence of postoperative FRI following open tibia fractures managed at a single National Health Service (NHS) Trust. 

Study objectives

The primary objective was to establish the proportion of patients who developed FRI within the study period. The secondary objectives were to examine whether the timing of intravenous antibiotic administration, the timing of initial surgical debridement, and the methods used for definitive skeletal fixation and soft-tissue management were associated with variations in infection occurrence. 

Hypothesis

The overarching hypothesis was that earlier administration of antibiotics and earlier initial debridement would be associated with a lower incidence of FRI in this patient cohort. 

## Materials and methods

Study site

This was a retrospective analysis of data done at a single UK NHS trust, including a Major Trauma Centre (MTC) and two District General Hospitals (DGH). 

Participants and sampling process

Patient sampling was done using the Trauma and Orthopaedic department’s Multidisciplinary Meeting (MDT) patient discussion list and the hospital's Electronic Patient Record (EPR) system. 

Eligibility criteria

Table 1 shows the inclusion and exclusion criteria used in the retrospective review. 

**Table 1:** Inclusion and exclusion criteria .

Inclusion criteria	Exclusion criteria
1. Age: all adult patients, aged 18 years and over 2. Gender: both male and female genders were included 3. Inclusion period: a five-year inclusion period between 1 January 2017 to 31 December 2022. 4. MDT discussion: all patients included were discussed in the Trauma and Orthopaedics morning MDT meeting.	1. Other open fractures: all patients with open fractures other than open tibia fractures 2. Limb amputation: all lower limb trauma calls which resulted in traumatic, unplanned amputations. 3. Conservative management: all patients were managed conservatively as their definitive management.

Primary and secondary outcomes

The primary outcome was to evaluate whether the timely administration of antibiotics affected the incidence of open tibia fractures. 

The secondary outcome was to determine whether delays in definitive management of soft tissues or skeletal stabilisation beyond 72 hours also resulted in higher rates of infection. 

Data collection

Initial data collection was carried out using the T&O department’s MDT handover Excel sheet. Search terms used in this local database included: “leg”, “tibia”, “open”, “tib”, “lower limb” and “open”, reflecting the terminology routinely used in the departmental handover rather than MeSH indexing. 

Further data confirmation was carried out using the hospital’s *report generator*, cross-checking all theatre databases for patients operated on within the inclusion period. 

The Trust’s Clinical Record System (CRS), known as *PowerChart*, allowed access to patient demographics, date and mechanism of injury, timing of antibiotic administration, time to theatre and operative notes. PowerChart was also used to access emergency department, admission, and discharge documentation, as well as clinic follow-up records. 

Data analysis

All data were entered into an Excel spreadsheet (Table 2). 

**Table 2:** Initial data collection on the Excel sheet: sections A-K. Image credit: Authors.

	Category	Data collected
A	Number	Patient identifier
B	Exclude	Transfer, other open fractures, paediatrics
C	Patient demographics	Age (years), gender, ethnicity
D	Dates and times	Date of injury, time of injury, time of arrival to emergency department, date of discharge, length of hospital stay (days)
E	Antibiotic administration	Antibiotics within three hours? Antibiotics used? Additional dose at definitive cover? Any missed doses?
F	Trauma call data	Trauma call done? Low or high energy mechanism? Mechanism? Contaminant? Neurovascular injury on admission?
G	Admission assessment	Time to splinting, type of dressing, type of splinting, pre-debridement photo
H	Operative procedure	Time to initial debridement, American Society of Anaesthesiologists classification, type of fixation, G&A classification, surgeon seniority, plastics presence in theatre and proforma used for documentation.
I	Definitive plastics procedure	Time from injury, type of closure and surgeon seniority
J	Complications and outcomes	Infection: pin site, superficial or deep. Micro-organisms isolated, nonunion, delayed union, malunion, delayed amputation, removal of metalwork, lost to follow-up.
K	Past medical history	Smoker, asthma, excess alcohol intake, chronic obstructive pulmonary disease, cancer, chronic kidney disease, cardiac, dementia, diabetes, hypertension, peripheral vascular disease, clotting disease, pregnancy and others

Data were collected in sections (from A to K) as follows: 

A. Number: Patient identifiers were initially collected in the hospital’s secure system and erased on completion to ensure confidentiality was maintained

B. Exclusion: Patients excluded from the study, including the reason for exclusion (transfer, other open fractures or paediatric age group).

C. Patient demographics: Including patient age, gender, and ethnicity.

D. Dates and times: Including time of injury, arrival at the emergency department, date of discharge and calculated length of hospital stay.

E. Antibiotic administration: Whether antibiotics were given within three hours of injury, the type administered, any additional doses given at initial debridement, and any missed doses. 

F. Trauma call data: Including mechanism of injury, any gross contamination from the external environment, and any neurovascular injury.

G. Admission assessment: Including time to splinting, type of dressing used, and clinical photography as per BOAST (British Orthopaedic Association Standards for Trauma) guidelines.

H. Operative procedure: Including time to initial debridement, American Society of Anaesthesiologists (ASA) score, type of fixation used (internal or external), trauma and orthopaedic surgeon seniority, plastic surgeon presence and seniority, and use of the BOAST proforma for open fractures. 

I. Definitive plastics procedure: Including time of definitive management and type of closure, such as split-thickness skin grafts (SSG) and local flaps. 

J. Complications and outcomes: Including the incidence of infections (deep, superficial and pin-site), types of microorganisms isolated from culture and sensitivity, and infections leading to delayed amputation or removal of metalwork.

K. Past medical history: Comorbidities such as diabetes, hypertension, chronic obstructive pulmonary disease (COPD) and smoking to evaluate whether they resulted in increased incidence of infection. 

Data were scored with a "0" if the answer was no and a "1" if the answer was yes. These scores were later summed.

Ethical considerations

A request for ethical approval was submitted to the Quality Improvement Projects (QIP) of the Trust before the commencement of data collection. Relevant guidelines and regulations were reviewed and taken into consideration during the data collection, analysis and write-up.

All patient identifiers were erased, and confidentiality was maintained.

## Results

Over the five-year inclusion period, a total of 440 patients presented to the Trust’s emergency departments, were trauma-called, and discussed in the T&O morning MDT meeting for having open tibia fractures. One hundred patients did not meet the inclusion criteria and were excluded from the study (58 were transferred from other hospital Trusts and 42 were younger than the included age group).

Overall, a total of 340 patients with open tibia fractures met the inclusion criteria and were further assessed. Of these, 33 (9.7%) patients developed open tibia FRIs. Table 3 shows the patient demographics. The mean patient age was 45 years, ranging from 21 to 85 years. 

**Table 3:** Patient demographics.

Total number of patients (meeting inclusion criteria)	340	
Total number infected	33	
Age (years)	Mean	45
	Minimum	21
	Maximum	85
Gender (number of patients)	Male	27
	Female	6

On average, patients who developed infections had longer hospital stays, remaining as inpatients for 20.85 days on average, ranging from 3 to 69 days.

The majority of patients, 32/33 (96.97%), received antibiotics promptly, within three hours of injury; however, 5/33 (15.15%) missed a dose while on the wards, as shown in Table 4.

**Table 4:** Administration of antibiotics.

Intervention	Number of patients
Antibiotics administered within three hours of injury	32
An additional dose of antibiotic administered on the induction of anaesthesia	31
Missed antibiotic doses	5

Higher-energy injuries were associated with a higher infection rate; 4/33 (12.1%) of patients with infections were exposed to farmyard contamination.

On average, the time to theatre was 21.66 hours post-injury. Initial debridement was performed for all patients, and the Gustilo-Anderson classification system was used, as shown in Table 5. 

**Table 5:** Gustilo and Anderson classification following initial debridement.

G&A type	Number of patients
Type I	2
Type II	2
Type IIIA	6
Type IIIB	9
Type IIIC	1
Not documented	13

**Figure 1:**
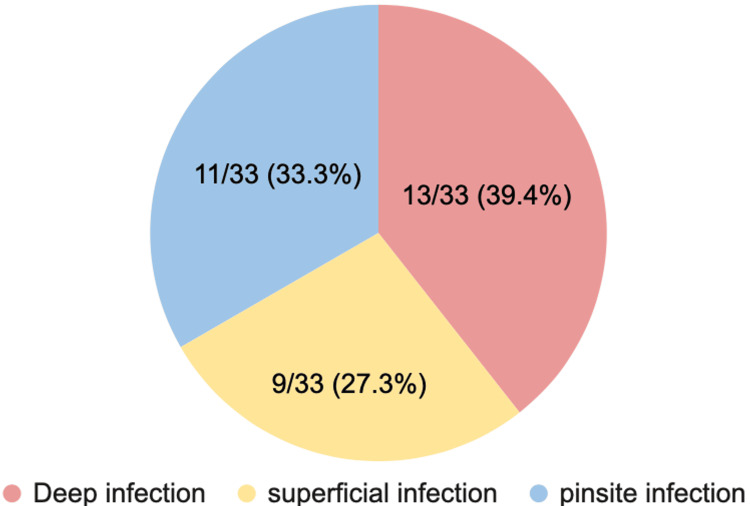
Incidence of infection severity. Image credit: Authors.

Of the 33 patients with infections, 9 (27.27%) had superficial skin infections and 13 (39.39%) had deep infections, of whom seven required further operative management for metalwork removal. Three patients underwent delayed limb amputation, and two were identified with infection-related non-union during subsequent clinic follow-ups. This is illustrated in Figure 1, which shows the severity of infections among the affected patients.

**Figure 2:**
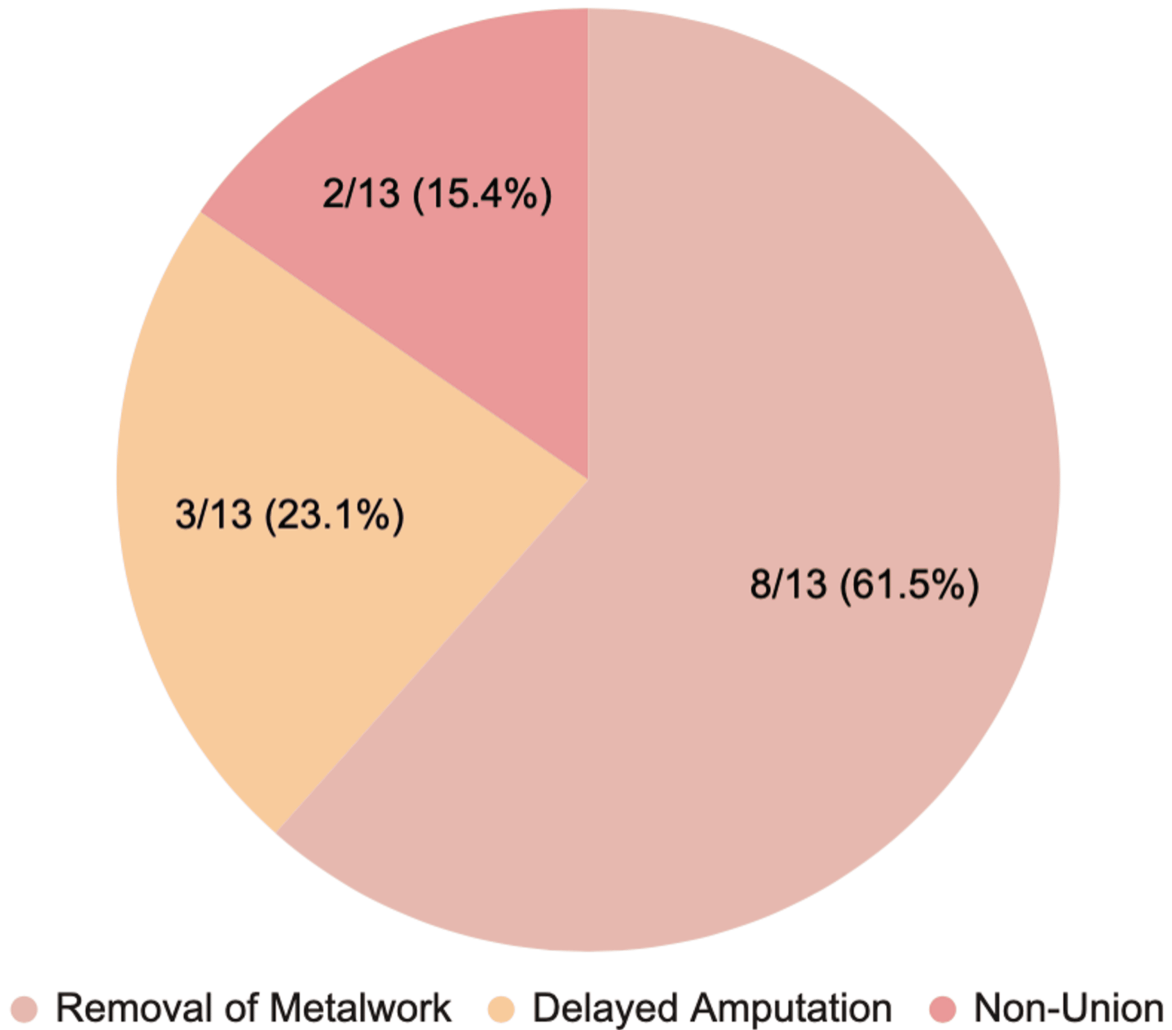
Outcomes of 13 patients with deep infections. Image credit: Authors.

Figure 2 shows the outcomes of deep infections, including metalwork removal, delayed amputation and non-union.

## Discussion

This five-year retrospective study evaluated FRI following open tibia fractures and explored whether early management factors, specifically antibiotic timing, timing of initial debridement and definitive fixation or soft tissue strategies, were associated with differences in infection rates. Of the 340 patients meeting the inclusion criteria, 33 (9.7%) developed an infection. This incidence falls within previously reported ranges for tibial fractures, where infection risk is known to be substantial, particularly in open fractures [1,4]. 

The high susceptibility of tibia fractures to infection is well established, with approximately 65% of FRIs occurring in the tibia due to limited soft tissue coverage and a high likelihood of contamination [3]. The severity of soft tissue involvement is a key determinant of infection, and the Gustilo-Anderson classification remains a widely used framework to describe the extent of injury [1]. Higher-grade injuries, particularly those involving extensive soft tissue damage or contamination, correlate with increased infection rates. Other classification systems, including the Orthopaedic Trauma Association system, further highlight the contribution of skin injury, muscle injury, arterial injury, contamination and bone loss to overall severity [6,7]. Historical FRI classifications, such as the Willenegger and Roth system, as well as more recent expert-derived classifications, similarly emphasise the relationship between complexity of injury and difficulty in eradicating infection [8]. These frameworks help contextualise the findings of this study, as a considerable proportion of infected cases involved in higher-grade or incompletely documented Gustilo-Anderson injuries. 

Reported incidence data from the United Kingdom indicate an annual open fracture incidence of 30.7 per 100,000 individuals, with open tibia fractures forming a significant proportion of this burden [1]. Deep infections in the lower limb can occur in up to 33% of severe injuries, reinforcing the need for rigorous early management to mitigate risk [9,10]. 

Risk factors for FRI can be grouped into patient-related, injury-related and treatment-related factors. Patient comorbidities such as diabetes, peripheral vascular disease and smoking have been shown to worsen outcomes in musculoskeletal infection [11,12]. Injury-related factors include energy mechanism and the presence of environmental contaminants, both of which were recorded in this cohort. Treatment-related factors, principally delays in antibiotics, delays in surgical debridement, method of fixation and duration of surgery, have also been implicated in infection development [11]. 

National and international guidelines emphasise the necessity of early intravenous antibiotic administration and timely debridement in the management of open fractures [13,14]. In this cohort, 96.9% of infected patients received antibiotics within three hours of injury; however, five patients had missed doses during their admission. Although this study does not allow causal inference, missed doses and delays in subsequent antibiotic administration are known to contribute to infection risk, and the presence of missed doses in infected cases highlights an important area for quality improvement. The average time to theatre among infected patients was 21.66 hours, which exceeds the early debridement intervals generally recommended in contemporary practice and may have contributed to the higher observed infection rate. External studies have demonstrated jay prolonged delays in debridement are associated with worse outcomes in open tibia injuries [15]. 

Microbiological findings from comparable UK major trauma centres demonstrate a predominance of Gram-positive organisms, especially *Staphylococcus aureus*, although mixed or Gram-negative infections are common in heavily contaminated injuries [16]. Studies from low-resource settings similarly describe high proportions of Gram-negative organisms, with comparable patterns of *S. aureus* dominance [17]. The data reinforce the importance of broad empirical antibiotic coverage and early administration, particularly in highly contaminated open fractures, and support the emphasis placed on antibiotic timing in early management pathways [18]. 

Integration of this study findings

The demographic profile of infected patients in this study aligns with published evidence: the majority were male, and the mean age was 45 years, consistent with populations sustaining higher-energy mechanisms [15]. The finding that nearly all infected patients received antibiotics within the three-hour window indicates that timing of the first dose alone may not fully account for infection development. Instead, the presence of missed doses in five patients may have contributed to inadequate antimicrobial coverage during the early post-injury period. 

Injury severity also played a notable role. A substantial proportion of infected cases were classified as Type IIIA and IIIB injuries, and a number lacked documented Gustilo-Anderson grading. Missing classification data limits the ability to fully stratify risk and reflects gaps in documentation that may influence care. The higher observed infection rate in cases requiring indirect soft tissue closure, such as vacuum dressings, split skin grafting or flap procedures, mirrors known associations between severe soft tissue loss, contamination and infection risk [3,10]. 

Similarly, the higher infection incidence among patients managed with external fixation is likely attributable to injury severity, rather than the fixation method itself. External fixations are commonly used in high-energy or contaminated fractures, and the need for staged procedures introduces multiple opportunities for bacterial colonisation. The outcomes observed in this study's deep infection cohort underscore the clinical significance of FRI: seven patients required removal of metalwork, three underwent delayed amputation, and two developed infection-related non-union. These findings echo the morbidity reported in other major trauma studies and highlight the long-term consequences of infection [16,17].

Study limitations

The COVID-19 pandemic overlapped with the study period and may have contributed to delays in emergency presentation, debridement timing and preoperative processes. As with all retrospective studies, the analysis is based on available documentation, which can vary in completeness, particularly in relation to classification recording and precise timing of interventions. Time to theatre data for non-infected patients were inconsistently documented in the retrospective dataset, preventing accurate calculation of a comparative mean value. These factors should be considered when interpreting the findings. Nevertheless, the study provides valuable insight into local practice patterns and highlights several areas within the early management pathway that may be amenable to optimisation. 

Recommendations

Improving the early management of open tibia fractures requires consistent adherence to established standards of care and accurate documentation of each step in the treatment pathway. In this cohort, incomplete documentation, particularly of classification grade, timing of debridement and antibiotic administration, limited the precision of data interpretation. Strengthening departmental familiarity with the open fracture documentation proforma and reinforcing its routine use may help ensure standardised assessment, improve adherence to recommended early management principles, and reduce the likelihood of missed or delayed interventions. 

A repeat five-year study with an inclusion period unaffected by the COVID-19 pandemic would enable more accurate evaluation of service performance by removing the confounding effects of system-wide delays. Such a study would also allow monitoring of pathway improvements implemented following this analysis. 

As this was a single-trust study, the findings reflect local practice patterns and may not be generalisable. A large multi-centre study within the United Kingdom is needed to evaluate prognostic factors across varied service models. This would help identify shared modifiable factors, support uniformity in pathway development and allow benchmarking across institutions. 

## Conclusions

In conclusion, based on five years of observation of FRI incidence in adult patients with open tibia fractures, adherence to BOAST guidelines is an essential step in preventing FRIs. Early antibiotic administration with no missed doses, prompt initial debridement, and timely definitive management were associated with the lowest infection rates. These findings support the hypothesis stated initially.
